# Food Advertising and Prevention of Childhood Obesity in Spain: Analysis of the Nutritional Value of the Products and Discursive Strategies Used in the Ads Most Viewed by Children from 2016 to 2018

**DOI:** 10.3390/nu11122873

**Published:** 2019-11-24

**Authors:** Mireia Montaña, Mònika Jiménez-Morales, Mercè Vàzquez

**Affiliations:** 1 Faculty of Information and Communication Sciences, Universitat Oberta de Catalunya, 08035 Barcelona, Spain; 2 Department of Communication, Pompeu Fabra University, 08018 Barcelona, Spain; monika.jimenez@upf.edu; 3 Faculty of Arts and Humanities, Universitat Oberta de Catalunya, 08035 Barcelona, Spain; mvazquezga@uoc.edu

**Keywords:** advertising, childhood obesity, food, health policy, language, nutrient profiling, television

## Abstract

In Spain, 40% of children are overweight or obese. Television advertising is a widely acknowledged factor contributing to high-calorie food intake. This study longitudinally correlates some variables involved in childhood obesity prevention strategies in Spain. A mixed-methods approach was used. A quantitative analysis of audience data was conducted to determine the advertising campaigns most viewed by Spanish children from 2016 to 2018. The Nutri-score system was applied to determine the nutritional quality of the food advertised. A content analysis and a study of the discursive strategies used as an advertising ploy was undertaken. The results were examined in relation to the regulatory framework of the Spanish PAOS Code for the co-regulation of food advertising aimed at children. The study shows that Spanish advertising aimed at children mostly advertises very low nutritional value products. Moreover, these campaigns violate the PAOS Code in terms of the use of language in relation to the product, its benefits, and the appearance of popular characters. Our findings suggest a direct association between low nutritional value food ads and discursive strategies based on the intangible and extrinsic characteristics of these products. There remains the need for stricter legislation that takes into consideration the nutritional value of advertised foods and the language used in their hedonistic advertising.

## 1. Introduction

Almost 40% of Spanish children are obese or overweight. According to the latest report from the World Health Organization (WHO), the prevalence of obesity among this group has grown alarmingly over the last decade in Spain, which currently has the highest rate of childhood obesity in Europe [1]. Among the causes of this increase, several studies point to the existence of an obesogenic context, which is the result of the relationship between television (TV) viewing and high-calorie food intake [2,3,4,5,6,7].

Despite the rise of new media and communication platforms, TV remains the most viewed communication media by children in Spain. According to data from the Spanish Media Research Association (AIMC, Asociación de Investigación en Medios de Comunicación) [8], 99% of children watch TV, and 80% do so daily. Consequently, TV is also the channel most widely used by advertisers to market food and beverages since it is highly effective among children, especially at the early ages [9,10].

In previous studies [11,12], it has been found that foods classified as processed, with low nutritional value due to their high sugar, fat, or salt content, dominate advertisements (ads) seen by children, with the result that there is greater TV exposure to food considered unhealthy than to healthy eating. Despite the poor nutritional value of the products advertised, most ads aimed at children make some type of nutritional or health claim [10], which misleads consumers who, believing in the healthy properties that the ad attributes to products with little nutritional value, end up consuming them [13,14].

In order to curb the statistical growth of overweight children, in 2005, the Spanish Agency for Consumer Affairs, Food Safety and Nutrition (AECOSAN, Agencia Española de Consumo, Seguridad Alimentaria y Nutrición) launched what is known as the NAOS Strategy (Strategy for Nutrition, Physical Activity and the Prevention of Obesity). The priority lines of action of the NAOS Strategy [15] include the goal of reducing the impact of food marketing on children through a self-regulating code of advertising. This goal was embodied in the PAOS Code [16], which, together with the Spanish Federation of Food and Beverage Industries, aims to co-regulate advertising messages targeted at children under the age of 12. The highlights of the PAOS Code refer to the presentation of the product, the language used for it, the benefits obtained, and the use of popular characters that act as prescribers in the purchase.

However, since it has come into force, several studies have highlighted the ineffectiveness of the PAOS Code. Research conducted by Romero-Fernández et al. [17] noted that almost 50% of ads from companies in the food and beverage industry that have signed up to the PAOS Code fail to comply with regulations. The research showed that non-compliance with the standards that could be quantified most objectively was very frequent.

Some authors have suggested that TV advertising of products with low nutritional quality should be restricted in order to reduce childhood obesity rates [18]. In this respect, a cross-sectional study compared levels of compliance with the PAOS Code during the year 2012 in relation to those from 2008 [19]. Non-compliance with the regulations increased and almost doubled in that period: 88.3% vs. 49.3%. The conclusions also underscored the ineffectiveness of the Code. In fact, the results of a study conducted in the US in 2009 had already shown that cases of childhood obesity can be reduced by up to 33% if TV advertising of unhealthy food is limited [20].

Common to the aforementioned studies is the fact that they point out non-compliance with the PAOS Code by advertisers with regards to product presentation and the use of characters popular among children. Both, specifically, are aspects in which minors are particularly vulnerable in terms of persuasive food marketing [21]. As several studies show, when food ads are viewed, areas of the brain are activated that are closely related to the purchasing decision process and subsequent consumption of the product. This effect is produced especially strongly when the message derived from the ad is associated with pleasure and rewarding experiences [22]. 

As Hyuksoo et al. [23] state, “it is also important to note that low-nutrition food commercials [use] both visual and audio special effects as peripheral cues more frequently than general-nutrition food commercials”. According to the author, children are susceptible to special effects, particularly when they are embedded in low-nutrition food ads. These effects might be particularly dangerous because children are attentive to these attention-getting techniques.

In fact, several studies have related the consumption of food with low nutritional value with low moods. Oliver, Wardle, and Gibson [24] refer to the concept of “emotional eaters” in relation to those people who seek to supplement their emotional needs through food intake. In this respect, “emotional eaters” consume more energy-dense foods with particularly low nutritional levels in response to negative emotions, compared to the rest of the population.

It is, therefore, no coincidence that most ads for processed foods with low nutritional value base their persuasive strategy on the promise of positive experiences.

In relation to this, it should be noted that previous studies conducted in Spain have already observed a form of ploy particularly harmful to children: associating the intake of a certain food with becoming the “best” [25]. Similarly, some emotional persuasive elements have been shown to have a very important role in the configuration of advertising messages [14,26]. In this regard, aspects such as pleasure, happiness, and hedonistic elements present in the advertising discourse of certain products positively impact consumption, since, by purchasing this product, people unconsciously seek to maintain the state of happiness promised by the advertising narrative [27]. In any case, these are strategies unrelated to the product itself, which, in the case of food products, often seek to mask their nutritional deficiencies [14,28]. 

Several studies have also found that that the effect is enhanced when the consumer empathizes with the characters that appear in the ad [29]. Using brand mascots and products [30], toy premiums [31,32], or characters known by the public also induce emotional responses that contribute to the effectiveness of the ads. According to Castonguay et al. [33], the combination of the use of characters known to children and using messages alluding to health and the excellent nutritional quality of products that, in reality, have a very low nutritional value is common and, at the same time, very harmful. Harris et al. [34] believe that marketing involving child-friendly cross-promotions (e.g., Nickelodeon, Disney Channel, and Marvel characters) accentuates the vulnerability of children to the advertising of low nutritional value products, undoubtedly contributing to obesity among that group.

Despite decades of research, children’s exposure to marketing of unhealthy foods seems to be an unsolved problem. As far as we know, previous research has focused only on children’s networks, not considering that child TV consumption involves all channels and schedules. Data from Kantar Media indicates that TV viewing by minors reaches its highest levels outside the legal time slot for the protection of minors. An average audience of 19% of this group was recorded—more than 1.2 million minor spectators—between 10 pm and 12 midnight (prime time); 5.4% of children also remained in front of the TV after midnight. This body states that this nocturnal child audience can be defined as inappropriate, undoubtedly due in part to the effect of certain programmes focused on families or even children that are broadcast during prime time and aired until the early hours of the morning on school days. This is something upon which not only parents should reflect, but also the television networks themselves, which must subordinate programming interests to societal wellness. 

The aim of this paper is to compare the nutritional value of the most viewed food ads by Spanish children, regardless of the time of broadcast or TV network, from 2016 to 2018, with the characteristics of the language used to present these products. We also analyze the presence of popular characters in the ads that encourage the purchase of the products. The results of the analysis are examined within the regulatory framework established in the PAOS strategy in order to determine the effectiveness of the regulations, especially among those advertisers who have publicly stated their adherence to the Code.

## 2. Materials and Methods

### 2.1. Material Design and Procedure

This cross-sectional study is designed based on a mixed-methods approach. A quantitative methodology was used to compile the audience data extracted from Kantar Media during the years 2016, 2017, and 2018. These data correspond to TV consumption by Spanish children aged 4 to 12 years during that period.

It should be noted that, unlike previous studies that have focused only on children’s networks, in this study, we analyzed the ads and audience of all the conventional TV channels that operate in Spain. The data obtained were not limited to the specific time slots for a child audience; instead, TV viewing by that group was considered at any time.

A qualitative analysis was carried out based on the components corresponding to the ads most viewed by the children’s group in question, captured in Infoadex Mosaico. This company monitors advertising activity in Spain. It has the largest database of the sector, classified with the highest level of detail and with quantitative data (insertions, investment, and occupation) and qualitative data (creatives). To that end, we selected the 100 most-viewed campaigns in each of the years analyzed, according to Kantar Media audience data. In total, we viewed and transcribed a total of 300 campaigns. We then searched all the ads corresponding to those campaigns, a total of 905, listed in Infoadex Mosaico, in order to perform a content analysis.

The analysis focuses on four research questions: First, what were the food ads most viewed by children aged 4 to 12 years old between 2016 and 2018, regardless of the time slot and network on which they were seen? Second, what was the nutritional quality of the advertised products most viewed by this target? Third, what kind of discourse was used in those campaigns? And finally, were there popular characters in those ads? The results obtained were compared with the regulatory framework defined by the PAOS Code in order to determine the effectiveness of the regulations, especially among those advertisers who have publicly stated their adherence to the code.

### 2.2. Nutritional Analysis

To perform this analysis, we classified the different products advertised, considering the following variables: food and nutritional value of each food. The nutritional value was calculated from Nutri-score, a system for assessing the quality of processed foods that allows them to be classified into colors, from red to green, like a traffic light. Studies such as Deschamps (2015) [35], among others, have validated the effectiveness of this system. France currently uses the Nutri-score system on its product labeling, while Belgium and Portugal are in the process of introducing it.

The Nutri-score system was approved in Spain at the end of November 2018. At present, adoption of the nutritional traffic light on labels by manufacturers is on a voluntary basis. Until 2020, it will not be compulsory for food to include the color scale resulting from the nutritional calculation carried out by Nutri-score on its labels. Given that the vast majority of the products examined did not have a nutritional label, in order to analyze the quality of the foods advertised, we applied the Nutri-score system to all the products that appeared in the processed food ads most viewed by Spanish children between 2016 and 2018.

### 2.3. Language Analysis

Based on the transcription of the 905 ads from the 300 food campaigns most viewed by children between 2016 and 2018, we analyzed the lexical items that are used in discourse content. We used the term lexical unit with the meaning of “a word taken in one well-specified sense and supplied with all the information specifying its behavior when it is used in this sense” [36]. 

Then, we classified these lexical items into different semantic fields; we grouped them into sets that share a common core of meaningful characteristics, in the same way that Fronzaroli (1993) defines a semantic field: “a group of words that stand in paradigmatic opposition to one another and share at least one semantic component” [37]. Analysis of the lexical items and their grouping into semantic fields allowed us to determine the type of discourse used in the presentation of food products in the ads most viewed by children. We also examined the relationship between the description of the food product by the advertiser and the intrinsic nutritional quality of that food.

The classification into semantic fields was specifically adapted to the typology of lexical items that were identified in each of the ads selected. Likewise, in the linguistic analysis, we took into consideration the use of a common linguistic resource, namely lexical creation, the aim of which is to capture the attention of the consumer and to present the product advertised in a more entertaining or attractive way.

To achieve the study objectives, the following variables were considered in the analysis of the lexical items: product, nutritional value, semantic field, and lexical items used in the ad.

### 2.4. Popular Character Analysis

For this point, we analyzed the presence of real, cartoon, or inanimate characters that are familiar to children due to their media presence. Based on this, we determined seven categories of “popular character”, as follows: communication professionals (journalists and TV presenters), actors and actresses, models, singers, athletes, chefs, and licensed film, TV, or comic characters.

### 2.5. PAOS Code Analysis

In this study, we examine different points of the PAOS Code in order to compare its regulatory framework with the content of the ads analyzed. In this respect, we focused on point 5 of section IV of the code, which refers to the product presentation; point 9 of section V, which refers to the language used in advertising aimed at minors; point 12 of section VI, relative to the benefits of the product; and point 14.1 of section VII, which focuses on the endorsement and promotion of the product through characters and programmes.

## 3. Results

### 3.1. Nutritional Analysis

The results revealed that from the 300 food campaigns most viewed by Spanish children aged 4 to 12 years between 2016 and 2018, only 8% corresponded to products with a high nutritional value (termed an “A” food nutritional label). A total of 19% of the foods had a medium-high value (B label), and 26% had a medium nutritional value (C label), while the nutritional value of 34% of the advertised foods was low (D label), and 13% was very low (E label). 

With regard to years, 8% of the products had a high nutritional green label (A label) in 2016, 9% in 2017, and 8% in 2018. In relation to products with a medium-high nutritional score that Nutri-score classified with a light green label (B label), we found that 20% of the foods advertised had a medium–high value in 2016, 16% in 2017, and 20% in 2018.

As the nutritional value of the products decreased, the number of products advertised increased: 30% of the advertised products in 2016 obtained the Nutri-score yellow label (C label). This figure decreased to 23% in 2017 and 25% in 2018. Additionally, during 2016, we found a total of 32 food products with a medium–low value, symbolized by the orange label (D label). This number increased to 36% in 2017 and 34% in 2018.

Most of the processed food products of the ads most viewed by Spanish children had a medium-low and low nutritional value. The red label (E) corresponded in 2016 to 10% of the products; in 2017, the figure grew to 16%, while in 2018 it fell again to 13% (Table 1).

### 3.2. Language Analysis

In order to determine the use of language in relation to the nutritional value of the advertised products, we carried out a linguistic study based on the message transmitted in the body of the ad. To that end, we analyzed the transcription of each ad and identified the lexical items central to each one; that is, the words that are significant or informative about the content of an ad.

Once the central lexical items of each ad had been identified, we classified them into a set of semantic fields, which showed a common core of meaningful features between these lexical items. The creation of these semantic fields allowed us to identify the most characteristic features of the foods from the different lexical items associated with them. We established a correlation between the nutritional value and the semantic field that described the product. We observed that the vast majority of the semantic fields most commonly used make direct reference to the qualities of the foods (e.g., *ser la leche* (creamy), *estar de miedo* (frightfully delicious), *ser la bomba* (great-tasting, nutty taste), *sin calorías* (calorie-free, no added sugar), *con vitamina B6* (contains vitamin B6), *único* (unique), *con una masa fina y crujiente* (thin crust)); a total of 294 adverts can be placed in this category.

Semantic fields related to moods derived from consuming the product (e.g., enjoy, take care of yourself, defeat, get, fight, smiles, surprise, pleasure, happiness) appeared in a total of 209 ads. Those related to foods themselves (e.g., turkey meat, homemade stew, eggs, sponge cake) were repeated in a total of 186 adverts. We also found a large number of ads (169) that included semantic fields related to leisure time (team, enjoyment, play, explore, collect, learn, travel, fun, superheroes, adventure), 100 with action (independence, discover, have power, learn, find out) and 53 related to meals (e.g., breakfast, afternoon snack, nibbles). We also observed that certain correlations occurred between semantic fields, such as between the semantic field of foods and the consumption of those foods during the different meals of the day (muffins and breakfast, chocolate and afternoon snack, cheeses, and dinner). These correlations allow the consumption of a certain product to be associated with a specific time of day, thus reinforcing the process of purchasing that product.

By linking these semantic fields to the nutritional quality of the products advertised, according to the Nutri-score system, we found that 61% of the cases that assessed semantic fields related to the nutritional quality of the product corresponded to foods from categories C, D, and E—low (chocolate products, high-fat and sugar yogurt, high-fat cheese, high-sugar cereals, ice-creams, ready meals), very low (industrial biscuits, pâtés, chocolate products, industrial pastries, high-sugar cereals, hot dogs), and little or no nutritional value (industrial bakery products and pastries, chocolate, sweets, mayonnaise, cured meats). The same percentage of cases, 61%, corresponded to the semantic field “moods”. The nutritional quality of the foods that use mainly semantic fields related with moods was also low (chocolate products, pizzas), very low (chocolate spread, biscuits), or practically non-existent (industrial bakery products and pastries).

Additionally, of the foods advertised in the 186 ads in which semantic fields associated with food predominate, 74% belong to nutritional categories C, D, and E. Seventy-six percent of the 169 adverts that used semantic fields related to “leisure time” advertised food products that belonged to the three lowest categories in terms of nutritional value according to Nutri-score. In relation to the semantic field “action”, in which we found a total of 100 ads, we observed that 74% of the products advertised also belonged to categories C, D, and E. Of the 53 cases that presented the concept “meals” as a semantic field, 79% of the products belonging to this category had low, very low, or practically no nutritional quality (Table 2).

### 3.3. Analysis of the Presence of Popular Characters

With respect to the appearance of popular characters in the ads analyzed, we found that this is a fairly commonly used resource in ads for processed food products (Table 3). A total of 49 known characters appeared in the 100 most viewed food campaigns during 2016. TV presenters appeared in a total of 15 campaigns. Likewise, in the ads from that year, 4 journalists, 15 actors, 4 singers, 6 athletes, 4 chefs, and 1 model also appeared. During 2017, the number of popular characters used in processed food advertising decreased significantly, with a total of 17 appearances: 3 presenters, 2 journalists, 6 actors, 2 singers, 1 athlete, 1 model, and 2 licensed film characters (Despicable Me and Star Wars). The last period analyzed, 2018, was the lowest of all. During this time period we found 1 TV presenter, 1 journalist, 5 actors, 1 singer, 1 chef, and 1 model—a total of 11 popular characters. The athletes who appeared most often were footballer Leo Messi, motorcyclist Marc Márquez, and swimmers Gemma Mengual and Mireia Belmonte, who are both Olympic medallists.

## 4. Discussion

The principal finding from this study is that the lower the nutritional value of the products advertised, the greater the presence of lexical items that transmit ideas related to positive experiences. Fun, happiness, adventure, and success were some of the concepts derived from the language used in the advertising of products with low nutritional value. These discursive strategies violate the regulatory framework established by the PAOS Code to prevent children obesity. The results of this study highlight clear non-compliance with several sections of the code. Thus, the most viewed ads by Spanish children largely corresponded to food products with low or no nutritional quality, which in no way helps to prevent obesity in this population. This supports the findings of previous studies [13,14] that have found that the PAOS Code does not regulate exposure to low-nutritional value product ads. Moreover, this kind of advertising was even more frequently played during child code-regulated broadcasts, and was reinforced during protected viewing time. However, the aforementioned studies did not analyze the advertising language used.

Additionally, the PAOS Code was also violated on point 5 section IV, considering the fact that the language used inferred acceptance, prestige, or the gaining of certain abilities through the acquisition and use of the product. In this sense, alluding to aspects such as strength, happiness, or victory was usually presented as a benefit or basic promise of the product in most of the campaigns.

The language used in the advertising of products with low-nutritional value undermine the Spanish Government’s strategy for the prevention of childhood obesity. It encourages children to consume these products, offering them positive emotions and experiences as a purchase benefit. Previous studies have proved that hedonic food advertising can alter the behavior of some people and can cause some disorders such as bingeing or phenomena related to the addiction of sensitization by incentives [38].

Although the PAOS Code regulates aspects linked to the advertising design of foods intended for children, these self-regulation guidelines do not take into consideration that the language used is often based on qualities extrinsic to the product, offering a positive emotional reward as a consumption value. Since children are a group who are especially sensitive to certain advertising incentives, this reward may be a highly influential factor in the buying decision process [21,22,23]. 

The discursive strategies used by these campaigns violate point 12 of section VI. This sets out that the benefits attributed to the food product must be inherent to their use. The advertisement must not give the impression that acquiring or consuming food products will make the child popular among his/her friends. The advertisements must not suggest that the purchase or use of the product would provide prestige, abilities, or any other special quality. In this sense, concepts such as “learn”, “be happy”, “be strong” or “defeat”, among others, can in no way be understood as benefits inherent to consumption of the product. Similarly, the use of these lexical items suggests acceptance and social recognition by purchasing the product. 

Point 14.1 of section VII of the PAOS Code recommends that in advertising aimed at children under 12 years of age, popular characters that may influence the purchase of the product should not appear. In spite of this fact, children night TV consumption is an access door to adult content. In this sense, most of the popular characters that appeared in the ads were from non-childhood professional fields, except for two chefs who appeared on a children’s programme and five well-known athletes. It is also notable that actors and singers who appeared in those ads were aimed at an adult audience as they appeared in late night content. Particularly significant was the low presence of characters from film productions aimed at children, and also of cartoon characters or mascots from certain brands that are especially popular among children. A high child audience during prime time, due to the broadcast of family programs, may be one of the factors that can propitiate it. It seems that advertising companies invest in prescribers that can impact the entire family unit, regardless of their age. Greater regulation of television programming during prime time would be desirable to decrease its viewing by children.

Although they may be questioned, government paternalistic restrictions on food and beverage options must address a significant health problem related to a specific type or class of food or beverages [39]. While voluntary regulatory codes from many countries (e.g., United Kingdom, Australia, New Zealand) have been ineffective in reducing exposures to non-healthy food advertising aimed to children [40,41,42,43], stricter restrictions (e.g., Quebec, Norway, Sweden) confirm that a ban on this kind of advertising can be effective in lowering non-healthy food consumption and increasing social welfare [44,45].

This research shows the need to relegate ads for processed products with low nutritional value to those time slots with a lower audience, and to emphasize self-efficacy and stricter regulation of advertising aimed at children [28,46] or for food advertising to be statutorily regulated, by banning the promotion of energy-dense, nutrient-poor (EDNP) products [13,47,48]. 

This study has many strengths and some limitations as well. To our knowledge, this research is the first to longitudinally correlate some of the variables involved in childhood obesity prevention strategies implemented in Spain, the advertising language, and the nutritional quality of products and relate them to the regulations themselves. It is interesting to note that this analysis of the semantic fields used in the discursive strategies is especially relevant, due to the impact that hedonistic advertising might have on food disorders. In this regard, we would like to highlight that the analysis carried out for this research focused on the most viewed ads by this group, regardless of whether they were seen during the time slot allocated specifically for children. Indeed, TV viewing by children reached its highest levels outside the legal time slot for the protection of children.

Regarding limitations, the present study naturally includes the fact that the research is specifically focused on TV advertising, while child media consumption covers a wide range of media, especially through digital devices. Thus, further studies on children’s exposure to digital food advertising are necessary. Another limitation would be that this research has focused on only one country, although this type of advertising could be representative of Western persuasive communication. In addition, this descriptive approach suffers from a lack of qualitative results based on child food preferences. Finally, an absence of quantitative data on child food consumption in Spain is another limitation of this research. 

## 5. Conclusions

This review indicates that the PAOS Code has consistent gaps. Aspects such as the nutritional value of the advertised food products should be considered. Despite most of the companies responsible for the analyzed campaigns supporting the PAOS Code and showing an initial commitment to respecting the regulatory framework contained therein, the code fails to regulate the high number of ads for products that do not contribute to the fight against childhood obesity. Our results demonstrate that there is a saturation of low-nutritional food ads compared to the virtual non-existence of healthy food campaigns. The language used in hedonistic advertisements of unhealthy food is a priority aspect to be treated. 

Finally, we consider that greater involvement of the Spanish food industry, as well as agencies and advertisers, is essential so that the self-regulation of advertising content is truly effective in the fight against childhood obesity. This measure would have the aim not only of protecting minors from ads for unhealthy food that are broadcast during prime time but also of showing consistency between reality and the general strategies for preventing obesity implemented by the Spanish Government. Future studies can fruitfully explore this issue further by comparing results from countries with different types of regulation.

## Figures and Tables

**Table 1:** Nutri-score classification of the 100 foods campaigns most viewed by children by year.

Food Nutritional Label	Food Campaigns *n* 2016 ^1^	Food Campaigns *n* 2017 ^2^	Food Campaigns *n* 2018 ^3^	Food Campaigns *n* (%) 2016–2018 ^4^
A	8	9	8	25 (8%)
B	20	16	20	56 (19%)
C	30	23	25	78 (26%)
D	32	36	34	102 (34%)
E	10	16	13	39 (13%)

^1^ Total *n* = 100; ^2^ Total *n* =100; ^3^ Total *n* = 100; ^4^ Total *n* = 300.

**Table 2:** Classification of the ads by semantic field.

Semantic Field	Ads n(%)
Food qualities	294 (32)
Moods	209 (23)
Foods	186 (21)
Leisure time	169 (19)
Action	100 (11)
Meals	53 (6)

Total *n* = 905.

**Table 3:** Presence of popular characters in the analysed campaigns.

Presence and Type of Popular Characters	Campaigns *n* 2016 ^1^	Campaigns *n* 2017 ^2^	Campaigns *n* 2018 ^3^
Non presence	51	83	89
Presence	49	17	11
Presenter	15	3	1
Journalist	4	2	1
Actor/Actress	15	6	5
Singer	4	2	1
Athlete	6	1	1
Chef	4	N/A	1
Model	1	1	1
Licensed character	N/A	2	N/A

^1^ Total *n* = 100; ^2^ Total *n* = 100; ^3^ Total *n* = 100.
